# Candidemia in Left Ventricular Assist Device Recipients: Incidence, Risk Factors, and Outcomes

**DOI:** 10.1093/ofid/ofaf251

**Published:** 2025-04-25

**Authors:** Rebecca Anderson, Stephanie Pouch, Lindsay Busch, Taylor Hayes, Susie Sennhauser, Joshua L Chan, Emily M Eichenberger

**Affiliations:** Emory University School of Medicine, Atlanta, Georgia, USA; Department of Medicine, Division of Infectious Disease, Emory University School of Medicine, Atlanta, Georgia, USA; Department of Medicine, Division of Infectious Disease, Emory University School of Medicine, Atlanta, Georgia, USA; Department of Medicine, Division of Infectious Disease, Emory University School of Medicine, Atlanta, Georgia, USA; Department of Medicine, Division of Cardiology, Emory University School of Medicine, Atlanta, Georgia, USA; Department of Surgery, Division of Cardiothoracic Surgery, Emory University School of Medicine, Atlanta, Georgia, USA; Department of Medicine, Division of Infectious Disease, Emory University School of Medicine, Atlanta, Georgia, USA

**Keywords:** bacteremia, candidemia, mechanical circulatory support, ventricular assist device, device endocarditis

## Abstract

**Background:**

Candidemia (*Candida* bloodstream infection [C-BSI]) in left ventricular assist device (LVAD) recipients is poorly understood. This study aimed to investigate the incidence, risk factors and outcomes of C-BSI in LVAD recipients.

**Methods:**

We screened 656 adults who underwent LVAD implantation at our institution from 1 January 2015 to 4 April 2024. Patients with C-BSI (n = 18) were compared with 2 control groups: (1) matched LVAD recipients with no bloodstream infection (N-BSI; matched 1:5; n = 90) to determine risk factors for C-BSI and (2) unmatched LVAD recipients with bacteremia (bacterial BSI [B-BSI]; n = 79) to compare mortality and infectious complication rates. A random forest model identified key predictive factors for C-BSI. Kaplan-Meier survival curves were used for time-to-event analyses.

**Results:**

Median time to C-BSI was 20 days after implantation (interquartile range, 6–42 days). Compared to N-BSI, C-BSI were more likely to require perioperative temporary mechanical circulatory support (9 patients [50%] vs 8 [8.9%], respectively), renal replacement therapy (12 [67%] vs 6 [6.7%]), total parenteral nutrition (6 [33%] vs 2 [2.2%]), and prolonged postoperative mechanical ventilation (for 12 days vs 1 day) (all *P* < .001). A random forest model identified ventilation duration, renal replacement therapy, and total parenteral nutrition as top predictors of C-BSI. In terms of outcomes, C-BSI was more likely to lead to device endocarditis than B-BSI (in 5 [28%] vs 7 [9.1%], respectively; *P* = .008) and was associated with shorter median survival after infection (25 [interquartile range, 12 to not estimable due to censoring] vs 490 [54 to not estimable due to censoring] days; *P* = .04).

**Conclusions:**

C-BSI occurs early in LVAD recipients and is associated with a high mortality rate. Identified risk factors identified may guide antifungal prophylaxis or early empiric antifungal treatment in this susceptible patient population.

## Teaser

Candidemia occurs early in LVAD recipients and is associated with high mortality. Recognized risk factors may guide targeted antifungal prophylaxis or help identify patients who could benefit from early empiric antifungal therapy at the first signs of sepsis.

Left ventricular assist devices (LVADs) are a form of durable mechanical circulatory support (MCS) used as both destination therapy and as a bridge to transplant in patients with advanced heart failure. Although the contemporary continuous-flow HeartMate 3 LVAD is associated with significantly lower rates of many adverse events seen with earlier devices, infection rates remain high, with up to 58% of HeartMate 3 recipients experiencing infection-related complications, such as local infections, driveline infections, and sepsis within 2 years of implantation [1].

Bloodstream infections (BSIs) are a particularly challenging complication in LVAD recipients and may or may not be device specific [2]. Device-specific BSIs involve the device components such as the driveline, cannula, or external surface of the implanted device whereas non–device-specific BSIs do not arise from the internally implanted or external transcutaneous portions of the MCS device [2]. Over time, these infections can evolve into device-specific BSI if ≥1 component of the LVAD becomes seeded by the BSI [3]. BSIs usually occur within the first 3 months of LVAD implantation, are often non–MCS related, and are associated with a significantly increased 2-year mortality rate [4].

While fungal infections have been shown to result in higher mortality rates and lower clearance of infection compared with bacterial infections [5], there has been limited research specifically focused on *Candida* BSI (C-BSI) in LVAD recipients. In the current study, we aimed to determine the incidence of C-BSI in LVAD recipients, identify risk factors for C-BSI by comparing LVAD recipients with C-BSI with those with no BSI (N-BSI), and examine infection-related complications, including endocarditis and death, by comparing LVAD recipients with C-BSI versus bacterial BSI (B-BSI).

## METHODS

### Study design

This is a retrospective case-control study of all adult patients who underwent LVAD implantation at a tertiary care academic hospital system between 1 January 2015 and 4 April 2024. LVAD recipients were identified from the LVAD database maintained by the LVAD center at Emory University and the Clinical Data Warehouse, a comprehensive repository integrating patient data, including laboratory results, medications, procedures and microbiologic reports. We screened all LVAD recipients for C-BSI and B-BSI using the microbiologic data from the Clinical Data Warehouse. These data were all verified through manual record review. LVAD recipients with C-BSI were compared with 2 control groups: (1) matched LVAD recipients without BSI (N-BSI), and (2) all LVAD recipients in whom bacteremia developed (B-BSI) (Supplementary Figure 1).

The primary aim of the C-BSI versus N-BSI comparison was to identify specific risk factors associated with candidemia in LVAD recipients. N-BSI was specifically defined as LVAD recipients who had no documented BSI, including patients in whom no blood cultures were obtained and those with negative blood cultures for the entire post–LVAD implantation period throughout our study. C-BSI cases were exactly matched without replacement 1:5 to N-BSI controls for sex and genetically matched using robust Mahalanobis distance for age at LVAD implantation. We used genetic matching with Mahalanobis distance for age to optimize covariate balance between groups and reduce bias in the study. Unlike traditional propensity matching methods, genetic matching iteratively selects the best matches by minimizing differences across multiple variables simultaneously, ensuring improved comparability between groups and minimizing confounding [6]. Matching for the C-BSI and N-BSI groups was performed using the *MatchIt* (version 4.5.5) R software package. The quality of matching was evaluated using the absolute standardized mean difference and empirical quantile-quantile plots.

The primary aim of the C-BSI versus B-BSI comparison was to evaluate infection-related consequences, including death and infective endocarditis. This comparison also allowed us to identify key differences in the source of infection and presenting symptoms in LVAD recipients with candidemia versus bacteremia.

For patients with B-BSI, coagulase-negative *Staphylococci* (except *Staphyloccus lugdunensis*), or *Bacillus* species found in only 1 of 2 blood culture sets were considered to be contaminants and were therefore excluded from the B-BSI group unless the infection was deemed a true BSI, as documented by the infectious disease specialist involved in the patient's care at the time of culture. If multiple episodes of B-BSI were documented in a patient, only the first episode was used for comparison with C-BSI. This study was approved by the Emory University Institutional Review Board (IRB; approval no. STUDY00006528); under this IRB protocol a complete waiver of HIPAA authorization and informed consent was granted.

### Definitions: Clinical Characteristics and Outcomes

Patient demographics (age, sex, and race) and microbiologic data were extracted from the Clinical Data Warehouse and confirmed with manual record review. Indication for LVAD, type of LVAD, medical comorbid conditions, perioperative medications, operative factors (ie, repeated surgical chest exploration and use of perioperative temporary MCS), infection characteristics (ie, symptoms, source of infection, and valvular or device endocarditis), and treatment details were extracted via manual record review. Outcomes of interest included device removal/exchange, heart transplantation, endocarditis, and time to death.

Return to the operating room was defined as repeat surgical chest exploration within 30 days of implantation. Use of perioperative temporary MCS included use of extracorporeal membrane oxygenation, right ventricular assist device, or biventricular assist device within 30 days before or after LVAD implantation. Line-associated infections were defined as BSIs specifically associated with central venous lines, in accordance with the National Healthcare Safety Network definitions [7]. Device-specific BSI was defined as positive peripheral blood cultures associated with positive cultures of external LVAD components and/or persistently positive cultures for ≥3 days. Device endocarditis was defined as positive peripheral blood cultures with radiographic evidence of endocarditis and/or the presence of septic emboli, in accordance with the International Society for Heart and Lung Transplantation 2024 infection definitions for durable and acute MCS devices [2].

Standard postoperative prophylaxis for patients undergoing LVAD implantation at our center during the study period included vancomycin plus cefuroxime or levofloxacin at the discretion of the implanting surgeon. No antifungal prophylaxis was used.

### Statistical Analysis

Statistical analysis was performed using R software (version 4.4.3). The *ggplot2* (version 3.5.1) package [8] was used to visualize data. Continuous variables are reported as median with interquartile range (IQR), and categorical variables are reported as frequency and percentage. Clinical characteristics were compared using Wilcoxon rank sum and Fisher exact tests, as appropriate. The cumulative incidence of candidemia was estimated using the competing risks method, with candidemia as the event of interest and death from any cause as the competing event. Cumulative incidence curves were generated using tidycmprsk [9] and ggsurvfit [10] packages, and the Gray test was used to compare the cumulative incidence between patients receiving implants in 2015–2019 versus 2020–2024. Time from LVAD implantation to infection and time from implantation to death or last follow-up were analyzed with Kaplan-Meier survival curves and compared using the log-rank test. Statistical significance was set at *P* < .05.

A random forest model was fit on the C-BSI and N-BSI data to identify the most important features predictive of C-BSI. A random forest model was selected over multivariable logistic regression given the small sample size and the random forest model's ability to capture complex, nonlinear relationships and interactions between variables. This model can perform well with small datasets because it reduces variance through averaging across multiple trees.

The data were first divided into a training and test set using a stratified random sample (80% training and 20% test). A validation set was derived from the training set to tune the hyperparameters. The validation set was created using a stratified split, maintaining 80% of training data for training and the remaining 20% for validation. A random forest model for classification was specified using the rand_forest() function from the *parsnip* (version 1.2.1) package [11]. Hyperparameters were set to tune the number of variables randomly sampled as candidates for each split and the minimum number of data points required to split a node. The total number of trees was set to 1000. The *ranger* [12] engine was used for model fitting. A grid of 25 combinations of the hyperparameters was evaluated on the validation set, and the model performance was evaluated using the area under the receiver operating characteristic curve (AUC). The best performing hyperparameters were selected based on the highest AUC score. The final model was fit using these hyperparameter values and evaluated on the hold-out test set. The top important features were identified using permutation importance.

## RESULTS

### Comparison 1: C-BSI Versus N-BSI

**Figure 1.**
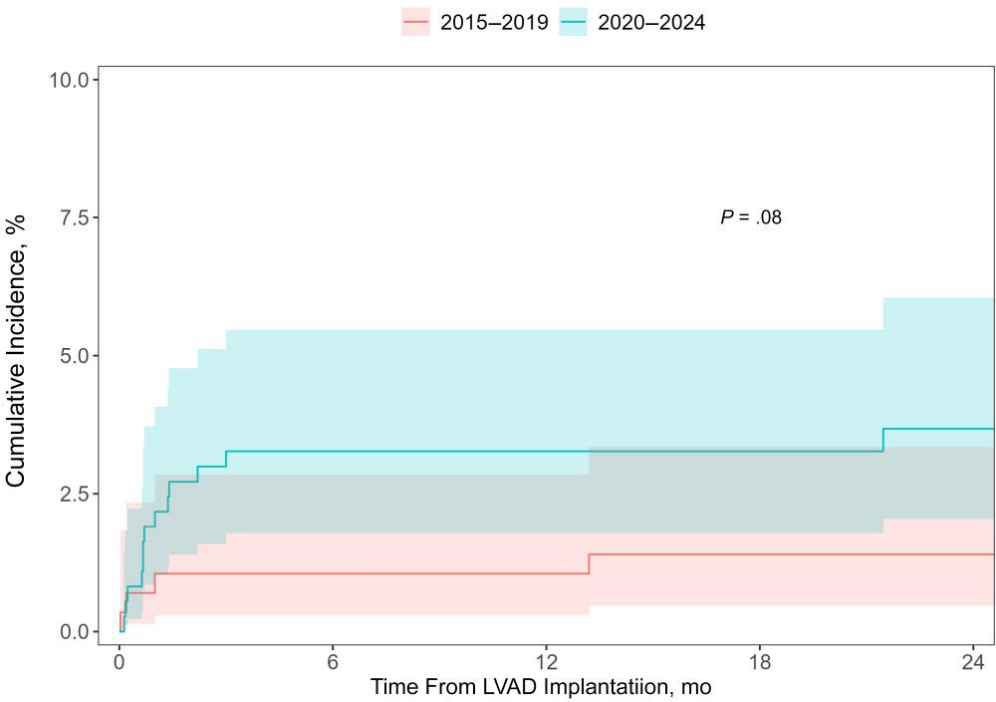
Cumulative incidence of candidemia by year of left ventricular assist device implantation.

Between 1 January 2015 and 4 April 2024, 656 patients underwent LVAD implantation at our center. C-BSI developed in 18 (2.7%) of LVAD recipients. The 1-year cumulative incidence of C-BSI was 1.0% (95% confidence interval, .29%–2.8%) for patients receiving implants in 2015–2019, compared with 3.3% (1.8%–5.5%) for those receiving implants in 2020–2024 (Gray test *P* = .08) (Figure 1).

Clinical and LVAD characteristics of the C-BSI versus N-BSI groups are provided in Table 1. Open chest after LVAD implantation (22% vs 5.6%, respectively; *P* = .04), return to the operating room for chest exploration (39% vs 13%; *P* = .04), and use of temporary MCS (50% vs 8.9%; *P* < .001) were significantly more common in the C-BSI than in the N-BSI group. There were no significant differences in the indication for LVAD or the type of LVAD implanted between the C-BSI and N-BSI groups. Patients who developed C-BSI were more likely than those with N-BSI to have received corticosteroids (50% vs 26% for N-BSI; *P* = .04) total parenteral nutrition (TPN; 33% vs 2.2%; *P* < .001), or continuous renal replacement therapy (RRT) (67% vs 6.7%; *P* < .001). Patients with C-BSI were more likely to have required a longer duration of mechanical ventilation after LVAD placement than those with N-BSI (median, 12 days vs 1 day, respectively; *P* < .001). History of tobacco use was less common in the C-BSI than in the N-BSI group (28% vs 56%, respectively; *P* = .03).

**Table 1.** Clinical and Infection Characteristics of Left Ventricular Assist Device Recipients With Candidemia, Bacteremia, or No Bloodstream Infection

Characteristic	Patients, No. (%)^a^		*P* Value^b^	Patients With B-BSI, No. (%)^a^ (n = 79)	*P* Value^c^
	No BSI, (n = 90)	C-BSI (n = 18)			
Age at VAD implantation, median (IQR),	43 (32–63)	46 (29–62)	.8	51 (41–60)	.5
BMI, median (IQR)^d^	28 (23– 33)	23 (21–29)	.08	29 (25–33)	.01
Sex					
Female	20 (22)	4 (22)	>.9	17 (22)	>.9
Male	70 (78)	14 (78)		62 (78)	
Race					
Black	58 (64)	12 (75)	.57	49 (62)	.40
White	32 (36)	4 (25)		30 (38)	
Missing	0	2		0	
Indication for VAD			.3		.3
Ischemic cardiomyopathy	21 (23)	2 (11)	.35	17 (22)	.51
Mixed ischemic/nonischemic cardiomyopathy	3 (3.3)	0 (0)	>.9	2 (2.5)	>.9
Myocarditis	0 (0)	1 (5.6)	.17	0 (0)	.19
Nonischemic cardiomyopathy not otherwise listed	56 (62)	14 (78)	.28	54 (68)	.57
Postpartum cardiomyopathy	9 (10)	1 (5.6)	>.9	6 (7.6)	>.9
Unknown	1 (1.1)	0	…	0	…
Type of VAD					
HeartMate II	2 (2.2)	0	>.9	7 (8.9)	.34
HeartMate 3	68 (76)	17 (94)	.11	49 (62)	.01
HeartWare	20 (22)	1 (5.6)	.19	23 (29)	.04
Open chest	5 (5.6)	4 (22)	.04	10 (13)	.3
Return to OR	13 (14)	7 (39)	.04	14 (18)	.06
Use of temporary MCS	8 (8.9)	9 (50)	<.001	14 (18)	.01
Time on ventilator after VAD placement, median (IQR), d	1.0 (1.0–2.0)	12.0 (4.5–17.5)	<.001	2 (1–4)	<.001
Use of TPN	2 (2.2)	6 (33)	<.001	4 (5.1)	.003
Corticosteroids within 30 d of VAD	23 (26)	9 (50)	.04	15 (19)	.01
RRT within 30 d after VAD	6 (6.7)	12 (67)	<.001	16 (21)	<.001
Diabetes mellitus	37 (41)	8 (44)	.8	36 (46)	>.9
HIV	0 (0)	1 (1.1)	>.9	1 (1.3)	>.9
History of tobacco use	50 (56)	5 (28)	.03	39 (49)	.1
Use of immunosuppressive medication	25 (28)	8 (44)	.2	15 (19)	.03
Prosthetic valve	5 (5.6)	2 (11)	.3	8 (10)	>.9
Fever	…	12 (67)	…	58 (73)	.6
Tachycardia	…	17 (94)	…	55 (70)	.04
Leukocytosis	…	17 (94)	…	66 (85)	.5
Hypotension	…	15 (83)	…	28 (36)	<.001
Driveline exit site infection with same bloodstream isolate	…	0 (0)	…	27 (35)	.003
Driveline exit site erythema or purulence during BSI	…	0 (0)	…	21 (27)	.01
Source of infection					
Driveline associated	…	0	…	20 (25)	.01
GI	…	0	…	5 (6.3)	
Line associated	…	6 (33)	…	10 (13)	
Mediastinitis	…	2 (11)	…	2 (2.5)	
Other	…	1 (5.6)	…	13 (16)	
Surgical site infection	…	0	…	2 (2.5)	
Unknown source	…	9 (50)	…	27 (34)	

Abbreviations: B-BSI, bacterial BSI; BMI, body mass index; BSI, bloodstream infection; C-BSI, *Candida* BSI; GI, gastrointestinal; HIV, human immunodeficiency virus; MCS, mechanical circulatory support; OR, operating room; RRT, renal replacement therapy; TPN, total parenteral nutrition; VAD, ventricular assist device.

^a^Data represent no. (%) of patients unless otherwise specified.

^b^ *P* values comparing patients with candidemia (C-BSI) and controls without BSI.

^c^ *P* values comparing patients with candidemia (C-BSI) and controls with bacteremia (B-BSI).

^d^BMI calculated as weight in kilograms divided by height in meters squared.

**Figure 2.**
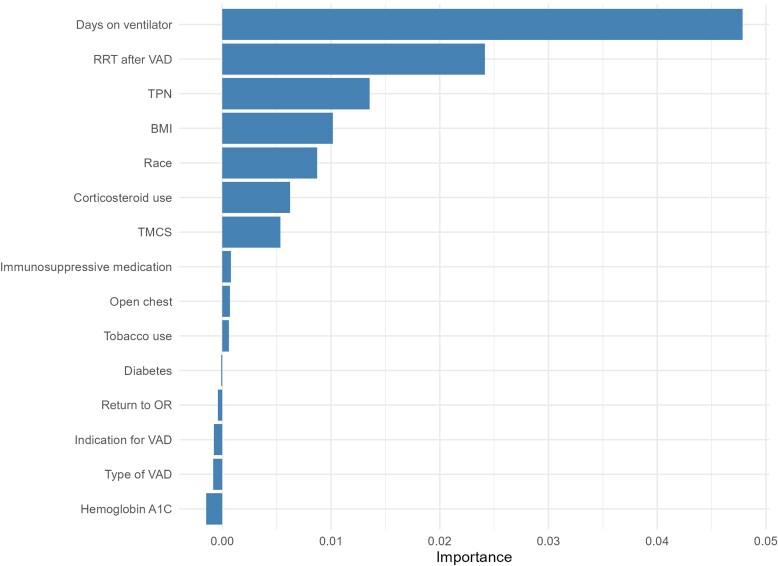
Variable importance factors for predicting *Candida* bloodstream infection (with a random forest model. The area under the receiver operating characteristic curve was 0.81. Abbreviations: BMI, body mass index; OR, operating room; RRT, renal replacement therapy; TMCS, temporary mechanical circulatory support; TPN, total parenteral nutrition; VAD, ventricular assist device.

A random forest model was used to identify the most important features predictive of C-BSI. Variable importance was assessed using permutation importance, which measures the decrease in model accuracy when the values of each variable are randomly shuffled. The model identified duration of mechanical ventilation, use of RRT after VAD placement, and use of TPN after VAD placement as the top 3 predictive features of C-BSI (Figure 2). The variable importance reflects how much a specific predictor contributes to the model's accuracy. An importance value of 0.048 (eg, the variable importance value for days on the ventilator) means that when the values of that variable are randomly shuffled—removing any meaningful relationship with the outcome—the model's accuracy decreases by 4.8%. This indicates that the variable explains approximately 4.8% of the model's predictive power. While the top variable only explains 4.8% of the model's predictive power, the model achieved an AUC of 0.81. This suggests that the other variables, even if individually less influential, collectively provide enough predictive information to provide strong classification performance.

**Figure 3.**
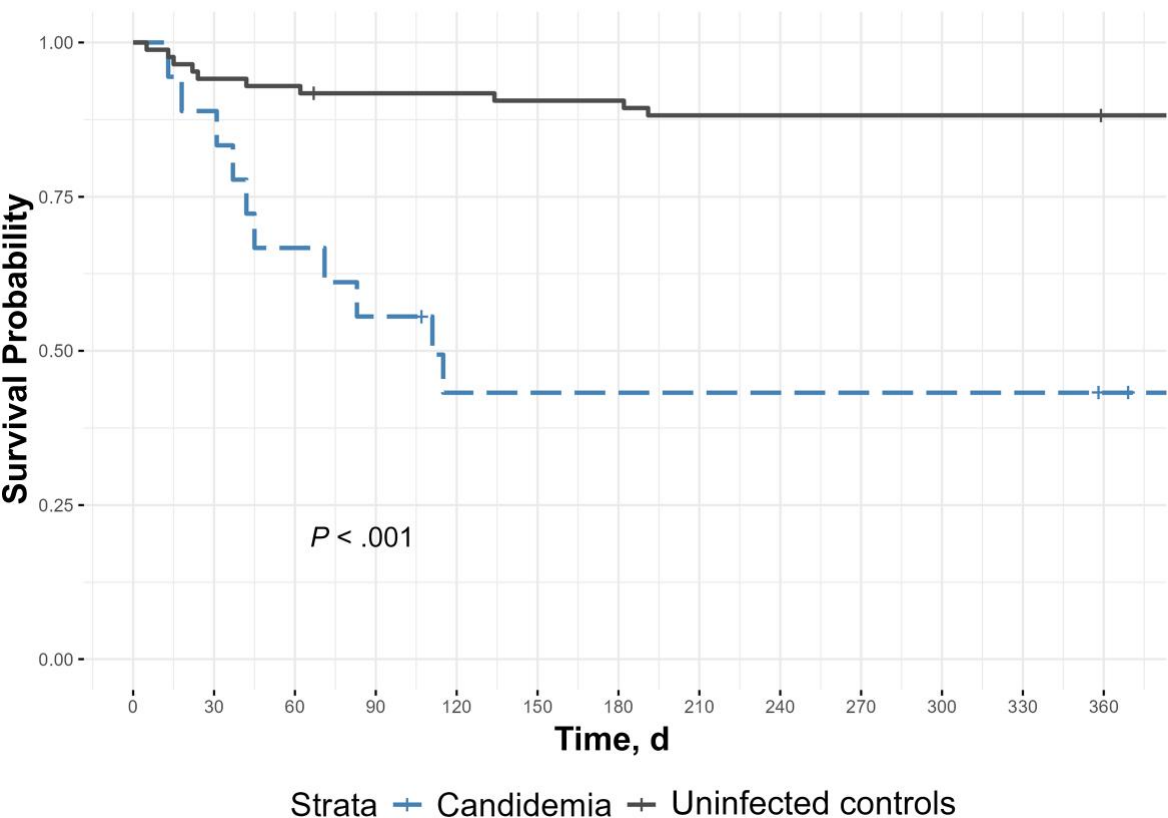
Time to death after left ventricular assist device implantation, comparing patients with *Candida* bloodstream infection (candidemia) and controls with no bloodstream infection.

When outcomes were compared, 18 patients (20%) in the N-BSI group ultimately received a heart transplant during the study period, compared with none in the C-BSI group (*P* = .02). The median time from LVAD placement to death was significantly shorter for C-BSI than for N-BSI (111 vs 2199 days, respectively; *P* < .001) (Figure 3).

### Comparison 2: C-BSI Versus B-BSI

During the study period, B-BSI developed in 79 LVAD recipients (12%). Peri-implantation temporary MCS was used more frequently in patients with C-BSI than in those with B-BSI (50% vs 18%, respectively; *P* = .01) (Table 1). The type of VAD differed significantly between C-BSI and B-BSI groups. Ninety-four percent in the C-BSI group had a HeartMate 3 versus 62% in the B-BSI group (*P* = .34); 0% versus 8.9% had a HeartMate II (*P* = .01); and 5.6% versus 29% had a HeartWare device (*P* = .04). Corticosteroid use within 30 days of LVAD implantation and use of immunosuppressive medications were more common among LVAD recipients with C-BSI than among those with B-BSI (50% vs 19% [*P* = .02] for corticosteroids and 44% vs 19% [*P* = .03] for immunosuppression). In the postoperative period, use of TPN and RRT were significantly more common in the C-BSI than in the B-BSI group (33% vs 5.1% [*P* = .003] for TPN and 67% vs 21% [*P* < .001] for RRT). Patients with C-BSI spent significantly longer on the ventilator after implantation (12 vs 2 days for B-BSI; *P* < .001). No patients had candidemia or bacteremia within the 7 days before LVAD implantation.

Among the 18 patients with C-BSI, *Candida albicans* was the most common species, isolated in 8 patients (44.4%), followed by *Candida parapsilosis* in 4 (22.2%), *Candida glabrata* and *Candida tropicalis,* in 2 patients each (11.1%), and *Candida krusei* and *Candida auris in* 1 patient each (5.6%). Five of the patients with C-BSI also had concurrent bacteremia (Supplementary Table 1). In the 79 patients with B-BSI, 22 unique bacterial organisms were identified. *Staphylococcus aureus* was the most commonly isolated bacteria in the B-BSI group, accounting for 30.4% of all cases (Supplementary Figure 2). At the time of infection, patients with C-BSI were more likely than those with B-BSI to be tachycardic and hypotensive (94% vs 70% [*P* = .04] for tachycardia and 83% vs 36% [*P* < .001] for hypotension). The source of infection significantly differed between the 2 groups (*P* = .01), with central line–associated infection (33% for C-BSI vs 13% for B-BSI) and infection from an unknown source (50% vs 34%) being more common in C-BSI. The median time from LVAD placement was 20 days (IQR 6–41) for C-BSI versus 293 days (51–814) for B-BSI. Eighty-nine percent of patients with C-BSI developed the BSI during the hospitalization for LVAD implantation.

In accordance with the 2024 International Society for Heart and Lung Transplantation MCS device infection definitions, we determined that 83% of C-BSIs were device specific versus 35% of B-BSIs (*P* = .001). In addition, device endocarditis was thought to be present in 28% of C-BSIs versus 9.1% of B-BSIs (*P* = .008). Native valve endocarditis was diagnosed in 17% of C-BSIs versus 3.9% of B-BSIs (*P* = .08). While 18% of patients with B-BSI eventually received a heart transplant over the study period, none of patients with C-BSI received one (*P* = .07).

**Figure 4.**
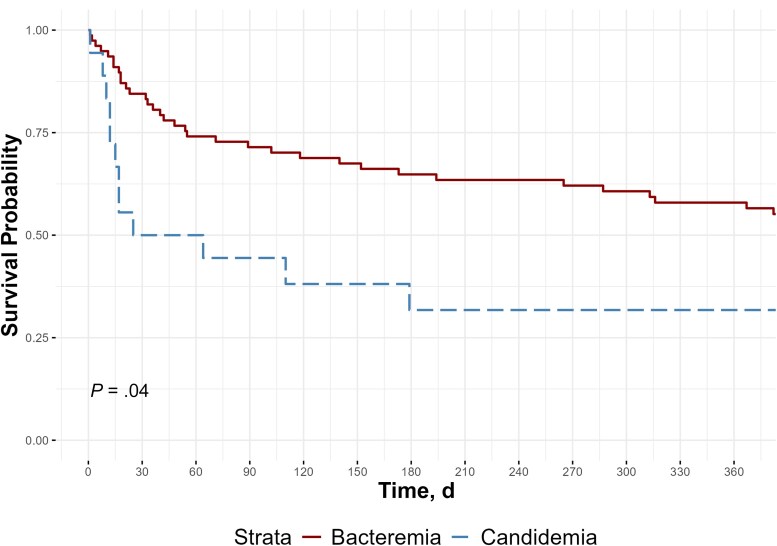
Time to death after first positive blood culture, comparing patients with *Candida* bloodstream infection (candidemia) and those with bacterial bloodstream infection (bacteremia).

The planned duration and actual duration of antifungal therapy of each patient with C-BSI are provided in Supplementary Table 1. Nine (50%) of the patients with C-BSI died before completing their course of antifungal therapy, and 22% did not have documented candidemia clearance at the time of death. One patient with C-BSI had blood cultures turn positive after death. Four patients were already receiving micafungin before collection of blood cultures. Among those not already receiving antifungals before culture collection, the median time to initiation of antifungal therapy was 1 day (range, 0–3 days). The time to death from the first positive culture was significantly shorter among for C-BSI than for B-BSI (median time to death [IQR], 25 [12 days to not estimable due to censoring] vs 490 days [54 to not estimable due to censoring]; log-rank *P* = .04) (Figure 4). Among patients with C-BSI, there was no significant difference in time to death between those with and those without concurrent bacteremia (log-rank *P* = .35).

## DISCUSSION

BSI is a common and severe complication occurring in LVAD recipients. In the current study, using retrospective data from a high-volume LVAD implantation center, we present one of the largest cohorts of LVAD recipients with C-BSI to date. Though uncommon, our study demonstrates that C-BSI is highly fatal among LVAD recipients. We found that the use of RRT after LVAD implantation, prolonged mechanical ventilation, and use of TPN after implantation were the top predictive risk factors for developing C-BSI [5, 9, 10]. The association of C-BSI with a substantially higher mortality risk relative to N-BSI and B-BSI in LVAD recipients underscores the need for proactive preventive strategies and rapid initiation of treatment for suspected C-BSI in patients at high risk.

The incidence of candidemia was 2.7% at our institution between 2015 and 2024, slightly higher than previously reported rates from other single-center studies (0.5%–1.7%) [5, 13, 14]. In comparison, the incidence of candidemia in critically ill patients without VADs has been reported to be 4.8–9.9/1000 intensive care unit admissions [15, 16]. The differences in the incidence of candidemia observed in our VAD population compared with that reported in the literature are not fully understood. We hypothesize it could be due to implantation of higher-acuity, more critically ill patients in recent years [17], though further research is needed to explore this possibility.

We found that use of temporary MCS, RRT, use of TPN, and longer duration of postimplantation mechanical ventilation are associated with candidemia in LVAD recipients. These findings are consistent with those of previous studies of candidemia, both in LVAD recipients specifically and in critically ill patients without an LVAD [4, 5, 18–21]. These interventions are likely linked to an increased fungal burden in critically ill patients and heightened risk of seeding intravascular catheters, biofilm formation, and candidemia [18].

Open chest after implantation and return to the operating room for additional chest operations were also associated with increased risk for C-BSI, as has been similarly noted in heart transplant recipients [22]. Open chest and repeated chest washouts after LVAD implantation may increase the risk of seeding the device with opportunistic pathogens such as *Candida*. The use of corticosteroids within 1 month of LVAD placement was also associated with candidemia in our cohort. Corticosteroids are known to contribute to dysregulation of the innate immune response to infection [19, 23, 24], and their use in patients with candidemia has been associated with increased mortality risk [19, 25]. While we identified several factors associated with C-BSI in our study, RRT, use of TPN, and longer duration of mechanical ventilation after implantation emerged as the top 3 predictors of C-BSI and may indicate those LVAD recipients at the highest risk for candidemia who may benefit from targeted antifungal prophylaxis after implantation or, alternatively, rapid initiation of empiric antifungals at the first signs of sepsis.

*C albicans* and *C glabrata* were responsible for most episodes of candidemia, similar to prior data reported by Aslam et al [5]. Our investigation detected a single case of *C auris,* which notably persisted for >4 months despite maximal medical therapy with 3 antimycotic agents. Alarmingly, other episodes of *C auris* BSI in LVAD recipients have been reported in the literature [26], underscoring the growing threat of this pathogen. The increasing incidence of *C auris* across the healthcare system [27] further confirms that it is an emerging and significant threat for LVAD recipients. *C auris* BSI in LVAD recipients represents a major challenge, given the ability of *C auris* to adhere to the hospital environment despite standard decontamination processes [28], its strong propensity for biofilm formation, and its well-documented resistance to fluconazole [29] and growing resistance to echinocandins [27].

In our study, 50% of C-BSI cases stemmed from an unknown primary source of infection. C-BSIs were more frequently device-specific infections and more likely to evolve into presumed device endocarditis than B-BSIs. We note that, in many cases, definitive diagnosis of device endocarditis [2] could not be made, particularly in a retrospective manner. Though ^18^F-fluorodeoxyglucose positron emission tomography/computed tomography (FDG PET/CT) has proved to be a valuable tool for identifying LVAD-specific infections [30], many patients with C-BSI in our cohort experienced rapid clinical deterioration and death, rendering inpatient FDG PET/CT impractical in this setting.

In our C-BSI group, the median time to death from infection was 25 days and crude mortality rate was 66.7% (12 of 18 patients). Other than a study conducted in Brazil with crude mortality rates as high as 76% in critically ill patients with candidemia [31], the crude mortality rate among LVAD recipients with candidemia in our study was higher than rates reported in critically ill patients without a VAD reported elsewhere [32, 33], and time to death was significantly shorter than in the B-BSI group. In addition, the incidence of device endocarditis was higher for C-BSI than for B-BSI. Current guidelines recommend managing *Candida* endocarditis in LVAD recipients with either liposomal amphotericin B and flucytosine or high-dose echinocandin followed by lifelong suppression in the event that the device cannot be removed [34, 35].

In our cohort, the first-line treatment for most episodes of C-BSI was micafungin followed by fluconazole for the remainder of treatment and lifetime suppression. Liposomal amphotericin B and flucytosine were often added in cases of persistent fungemia. Despite general adherence to existing treatment guidelines, 50% of patients with C-BSI died before completing therapy, and 22% did not have documented clearance of *Candida* from the bloodstream at the time of their death. This emphasizes that medical therapies, while necessary, are often insufficient in clearing C-BSI.

In addition to choosing the appropriate antifungal regimen, source control (ie, removal of infected foreign material) is another major pillar of treating C-BSI. For example, removal of central venous catheters is associated with reduced mortality rates in patients with invasive candidiasis [36]. While *Candida* species have the propensity to form biofilms and seed foreign devices, patients with C-BSI are often hemodynamically unstable, making LVAD explantation or exchange prohibitive. In our study, none of the patients with C-BSI and 6.5% of those with B-BSI underwent LVAD exchange.

While a systematic review found that pump exchanges offered no mortality benefit or decreased incidence of recurrent infections in those with LVAD infections [37], it is important to note that the presence of an indwelling MCS device exists as a persistent nidus of infection. The risk of device exchange in these cases, therefore, must be weighed carefully against risks of medical therapy alone. In the setting of organ nonrecovery, a heart transplant is the only definitive cure for LVAD with C-BSI [3]. Unfortunately, none of the patients with C-BSI received a heart transplant during the study period. While LVAD infection, including recent BSI, is not a contraindication for heart transplantation, patients with active BSI or septic shock are likely too high risk for a heart transplant [3].

With limited success in treating C-BSI in LVAD recipients, prevention remains critical. General recommendations on the use of anti-*Candida* prophylaxis and empiric treatment are variable across clinical practice guidelines [38]. The Infectious Diseases Society of America 2016 guidelines recommend antifungal prophylaxis only in patient groups determined to be at high risk with a rate >5% for invasive candidiasis [34], though these guidelines do not specifically comment on the use of antifungal prophylaxis in all LVAD recipients [34], and the practice appears to be institution dependent. Aslam et al [5] found that antifungal preoperative prophylaxis with fluconazole was unrelated to the subsequent development of fungal VAD infections but commented that it is reasonable to study the use of extended antifungal prophylaxis in VAD recipients until removal of invasive devices or to investigate alternative prophylaxis medications, such as echinocandins.

Regarding empiric treatment, both the Infectious Diseases Society of America and the European Confederation for Medical Mycology guidelines recommend initiating empiric antifungal treatment in patients deemed at risk for invasive candidiasis, including those with septic shock, prolonged intensive care unit stays, and indwelling central venous catheters [34, 35]. One study found that empiric systemic antifungal therapy in critically ill patients led to similar 28-day mortality rates as those in patients not treated with systemic antifungal therapy, despite the former group being more critically ill [39].

The potential benefit of anti-*Candida* prophylaxis and/or early empiric treatment must be carefully weighed against the risks, particularly the development of resistance to antifungal therapies [40]. Although the incidence of C-BSI seen in our study was <5%, we emphasize the importance of risk stratification within the LVAD population. C-BSI occurs early, is highly fatal, and poses significant challenges for surgical source control, which for full eradication would require LVAD removal/exchange or heart transplantation. The factors associated with C-BSI identified in this study, such as the use of RRT, TPN, and prolonged mechanical ventilation, align with established risk factors for C-BSI in critically ill patients without an LVAD.

Our findings highlight an opportunity to more effectively identify at-risk LVAD recipients, potentially enabling more targeted prophylaxis or early empiric treatment when certain risk factors are present. For example, antifungal prophylaxis may be explored for LVAD recipients who require RRT or TPN or remain ventilator dependent for beyond the first week after implantation. However, based on our study, it is not possible to establish a specific cutoff for the duration of mechanical ventilation that would warrant antifungal prophylaxis. Alternatively, LVAD recipients exhibiting these risk factors may benefit from early initiation of empiric antifungal treatment at the first sign of sepsis. We encourage other centers to report their experience with C-BSI in the LVAD population to better elucidate effective prevention strategies and improve outcomes for those affected by this infection.

Our study has limitations. First, this is a single-center retrospective study. Small sample size may have restricted our capacity to detect all statistically significant differences between groups. B-BSIs were classified appropriately to the best of our ability; however, some cases may have been missed in the microbial data acquisition process due to transition to a new electronic medical record system after 2022 and changes in microbial reporting. Finally, comparing our C-BSI rates in LVAD recipients with those in critically ill patients without LVADs at our institution would have been an interesting addition to the study, but these data were not available. We encourage other centers to examine this comparison as well as to report on their experience managing C-BSI in LVAD recipients.

In conclusion, we have demonstrated that C-BSIs occur early in a patient's postoperative course, are frequently device specific, and are often associated with rapid clinical decompensation and death. These findings, in combination with the notable contributing risk factors identified in our study, support consideration of antifungal prophylaxis or rapid initiation of empiric antifungal therapy in specific at-risk groups.

## Supplementary Material

ofaf251_Supplementary_Data

## Supplementary Data

Supplementary materials are available at *Open Forum Infectious Diseases* online. Consisting of data provided by the authors to benefit the reader, the posted materials are not copyedited and are the sole responsibility of the authors, so questions or comments should be addressed to the corresponding author.

## Notes

***Data availability.*** Data are available from the authors on request.

***Financial support*.** This work was supported by the National Institutes of Health (grant 1K23AI175676-01A1 to E. M. E.), the National Center for Advancing Translational Sciences of the National Institutes of Health (grants UL1TR002378 and KL2TR002381 to J. L. C.), and the Carlyle Fraser Heart Center (support to J. L. C.).
