# Supplementary material for: Candidemia in Left Ventricular Assist Device Recipients: Incidence, Risk Factors, and Outcomes
Source: Open Forum Infect Dis. 2025 Apr 25;12(5):ofaf251. doi: 10.1093/ofid/ofaf251 (PMC12079779; doi:10.1093/ofid/ofaf251)
Supplement: ofaf251_Supplementary_Data [file ofaf251_supplementary_data.docx]

**Supplemental Table 1:**

|  | | Supplemental Table 1: Microbiology, management and outcomes in 18 cases of candidemia | | | | | |
| --- | --- | --- | --- | --- | --- | --- | --- |
| Patient | **Candida species** | | **Concurrent bacteremia?** | **C-BSI during VAD**  **implant**  **hospitalization?*** | **Persistent fungemia?**** | **Management of Candidemia**  **(Actual duration of treatment/intended treatment days)** | **Outcome** |
| 1 | *C. parapsilosis* | | - | yes | no | Treatment: Micafungin (14/14)  Suppression: none | Cultures cleared; patient transferred care |
| 2 | *C. albicans* | | - | yes | no | Treatment: Micafungin > fluconazole (42/42) Suppression: fluconazole | Cultures cleared; patient died on suppression |
| 3 | *C. albicans* | | - | yes | no | Treatment: micafungin (14/14)  Suppression:  none | Cultures cleared;  Patient living |
| 4 | *C. parapsilosis* | | - | yes | yes | Treatment: micafungin > LAMB/flucytosine>micafungin/fluconazole  (42/42)  Suppression: fluconazole | Cultures cleared; patient living |
| 5 | *C. tropicalis* | | - | yes | no | Treatment: n/a  Suppression: n/a | Patient died before being started on therapy |
| 6 | *C. albicans* | | - | yes | yes | Treatment: micafungin/LAMB/flucytosine  Suppression: n/a | Cultures cleared; patient died on therapy |
| 7 | *C. glabrata* | | *Rothia mucilaginosa* | yes | no | Treatment: micafungin (30/30)  Suppression: none | Cultures cleared; patient died after completing therapy |
| 8 | *C. albicans* | | - | yes | no | Treatment: micafungin (42/42)  Suppression: fluconazole | Cultures cleared; patient living |
| 9 | *C. albicans* | | - | yes | no | Treatment: micafungin (10/42)  Suppression: n/a | Cultures cleared; Patient died on therapy |
| 10 | *C. tropicalis* | | *Bacteroides thetaiotaomicron* | yes | yes | Treatment: micafungin > fluconazole > micafungin > lamb/flucytotine (20/***)  Suppression: n/a | Cultures cleared; Patient died on therapy |
| 11 | *C. parapsilosis* | | - | yes | yes | Treatment: micafungin >LAMB/flucytosine>micafungin>fluconazole (60/60)  Suppression: fluconazole | Cultures cleared; Patient living |
| 12 | *C. auris* | | *Escherichia coli* & *Enterococcus faecium* | yes | yes | Treatment: LAMB/flucytosine/isavuconazole (indefinite) Suppression: n/a | Persistently candidemic; patient stable on treatment |
| 13 | *C. glabrata* | | - | yes | yes | Treatment: micafungin/LAMB>fluconazole (24/***)  Suppression: n/a | Cultures cleared; Patient died on therapy |
| 14 | *C. parapsilosis* | | - | yes | - | Treatment: LAMB> fluconazole (9/***)  Suppression: n/a | Unclear culture clearance; Patient died on therapy |
| 15 | *C. albicans* | | - | no | yes | Treatment: micafungin (10/42) Suppression: | Cultures cleared; Patient died on therapy |
| 16 | *C. albicans* | | - | no | no | Treatment: micafungin (30/30)  Suppression: fluconazole | Cultures cleared; Patient transitioned to hospice |
| 17 | *C. krusei* | | *Escherichia coli* | yes | yes | Treatment: micafungin. Micafungin/voriconazole (9/***)  Suppression: n/a | Cultures positive; Patient died on therapy |
| 18 | *C. albicans* | | *Escherichia coli* | yes | no | Treatment: micafungin> fluconazole (11/16)  Suppression: n/a | Cultures cleared; Patient died on therapy |
| *First episode of candidemia occurred in the same hospitalization as VAD implantation  **Persistent candidemia defined as positive blood cultures for ≥ 72 hours  ***Duration of treatment not clearly defined in infectious disease clinical notes  BSI; bloodstream infection, LAMB; liposomal amphotericin B | | | | | | | |

**Supplemental Figure 1: Consort Diagram**

LVAD recipients implanted at Emory between 1/1/2015-4/4/2024 were screened for development of bloodstream infection. Among those who developed a bloodstream infection, patients were divided into two groups: those who developed candidemia (C-BSI) and those who developed bacteremia (B-BSI). C-BSI were matched 1:5 to patients with no documented bloodstream infection on age and sex to generate a matched control group (N-BSI). Comparison 1 compared C-BSI to N-BSI to determine risk factors for C-BSI in LVAD recipients. Comparison 2 compared C-BSI to B-BSI to understand how having *Candida* bloodstream infection impacts outcomes in LVAD recipients relative to bacterial bloodstream infections.

**
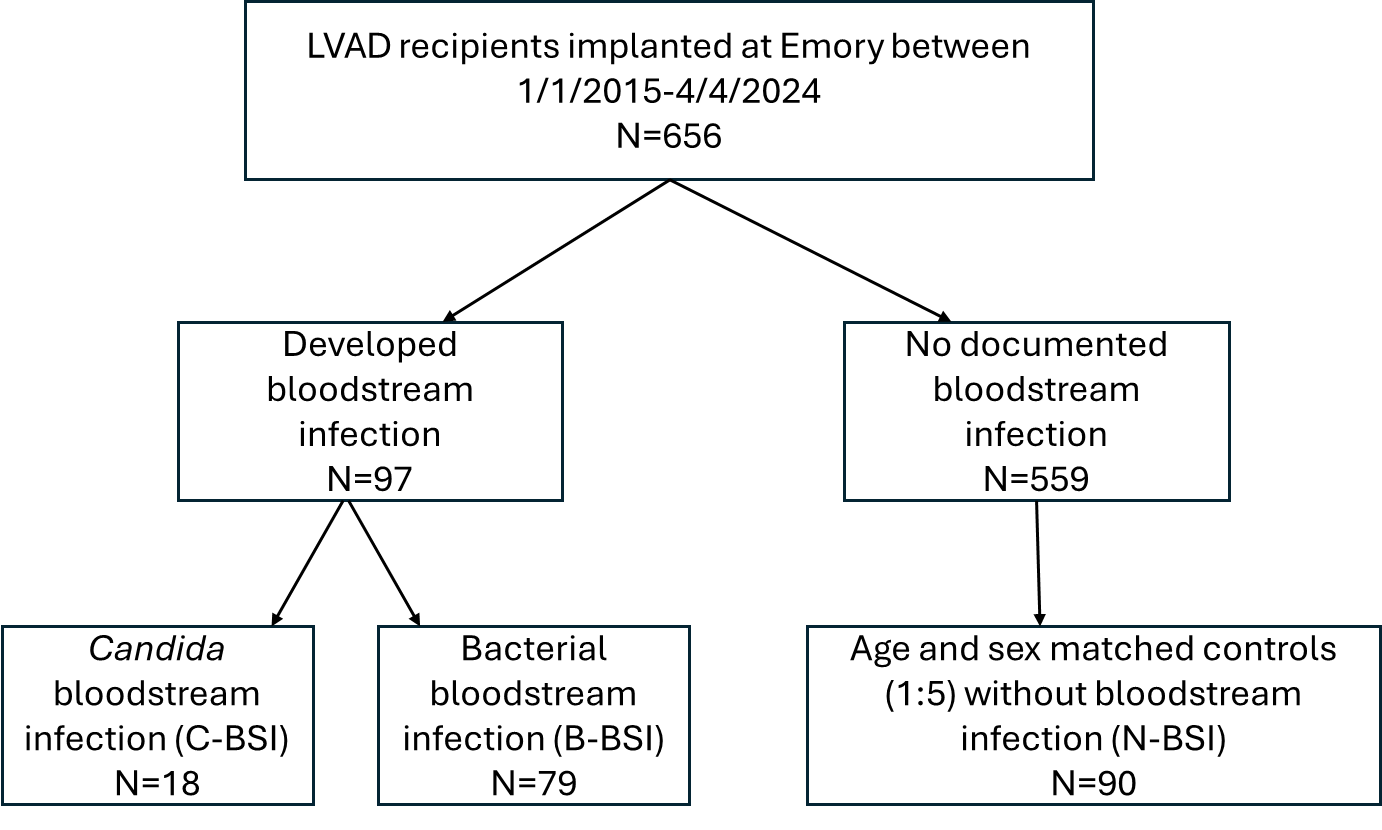
**

**Supplemental Figure 2: Causative organism of bacteremia**

Spp, species; B-BSI, bacterial bloodstream infection.

**
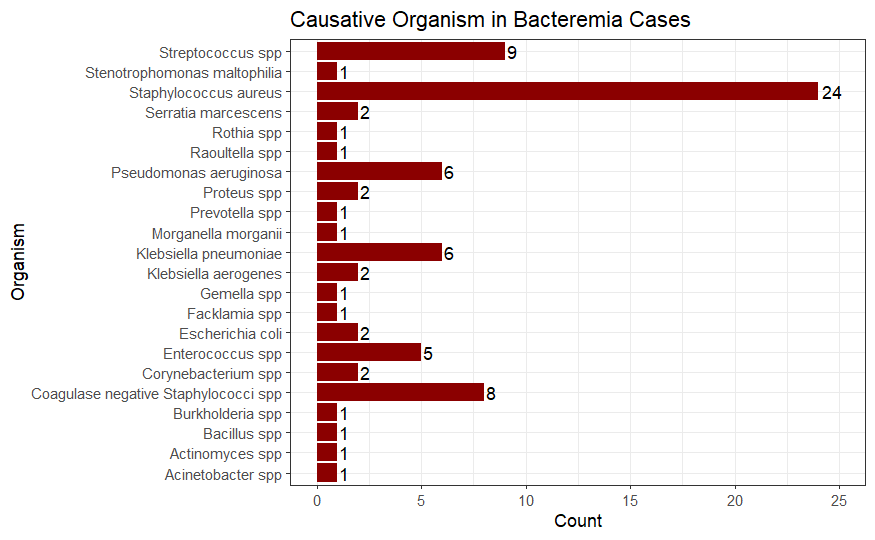
**
